# Bilobed flap technique as alternative for patient with eccrine spiradenoma: A rare case report

**DOI:** 10.1016/j.ijscr.2023.108549

**Published:** 2023-07-21

**Authors:** Muhammad David Perdana Putra, Gumilar Fardhani Ami Putra, Widyanti Soewoto, Kristanto Yuli Yarso, Galih Aktama, Shinta Andi Sarasati

**Affiliations:** a Department of Surgery, Universitas Sebelas Maret, Indonesia; b Master Program in Biomedical Science, Universitas Brawijaya, Indonesia; c Department of Surgery, Oncology Surgery Subdivision, Universitas Sebelas Maret, Indonesia; d Department of Surgery, Universitas Sebelas Maret, Indonesia; e Department of Pathological Anatomy, Universitas Gadjah Mada, Indonesia

**Keywords:** Eccrine spiradenoma, Facial, Bilobed flap, Adnexal tumor

## Abstract

**Introduction and importance of case:**

Eccrine spiradenoma (ES) is a rare benign skin adnexal tumor. ES resembles many other dermal lesions and is commonly found at the face. Hence, proper diagnosis and treatment with aesthetic considerations are needed.

**Case presentation:**

Here we reported a case of a 47 years old male who came with a mole-like mass on the lower side of the left eye socket. The histopathological examination showed small basaloid cells surrounding the larger, paler epithelial cells, a characteristic feature of spiradenoma. The surgery was then performed using bilobed flap technique, considering that the pathological mass was oval-shaped.

**Clinical discussion:**

Bilobed flap technique preserves skin color and texture, providing excellent tissue match and minimizes donor site complication. This technique distributes skin tension along a wider, curved area of the site.

**Conclusion:**

Bilobed flap technique can be used in round or oval-shaped facial tumor such as ES.

## Highlights

• Eccrine spiradenoma is a benign adnexal tumor commonly occurring in the facial region.
• Eccrine spiradenoma resembles many other dermal lesions, requiring biopsy for proper identification.
• The cosmetic aspect is important in the management of eccrine spiradenoma.
• The bilobed flap resulted in good cosmetics and minimal complication, showing good potential for eccrine spiradenoma.

## 1 Introduction

ES is a benign adnexal tumor arising from the intradermal ducts of eccrine glands. ES is rare, with only 50 cases reported [1]. ES predominantly affects the middle-aged. It can resemble many other dermal lesions, hence biopsy is essential for the proper identification of ES. It is important to note that a malignant version of ES also exists with a 50 % metastatic rate and 37 % mortality rate, making early diagnosis important. The transformation to malignancy can be found in a prolonged benign lesion [2].

This case has been reported in line with the SCARE criteria [3].

## 2 Case presentation

**Fig. 1:**
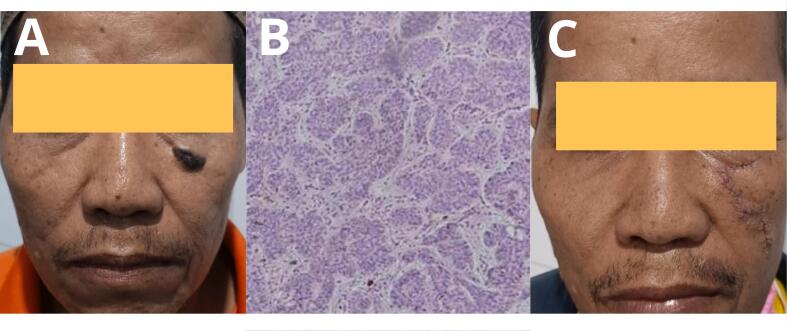
(A) Clinical appearance of the patient before surgery. (B) Histological analysis showing encapsulation by basaloid cells. (C) After surgery.

A 47-year-old man presented with a complaint of a mole-like lump on the left lower part of the eye socket. There was no family history with similar complaint. The surface of the tumor mass was uneven, measuring 1.2 cm × 1 cm. There was no bleeding or discharge. Histopathological analysis showed small basaloid cells surrounding larger, paler epithelial cells, typical of a spiradenoma. In this patient, a small ductal lumen can be seen in the center of the lobe, with no cellular pleomorphism found (Fig. 1).

**Fig. 2:**
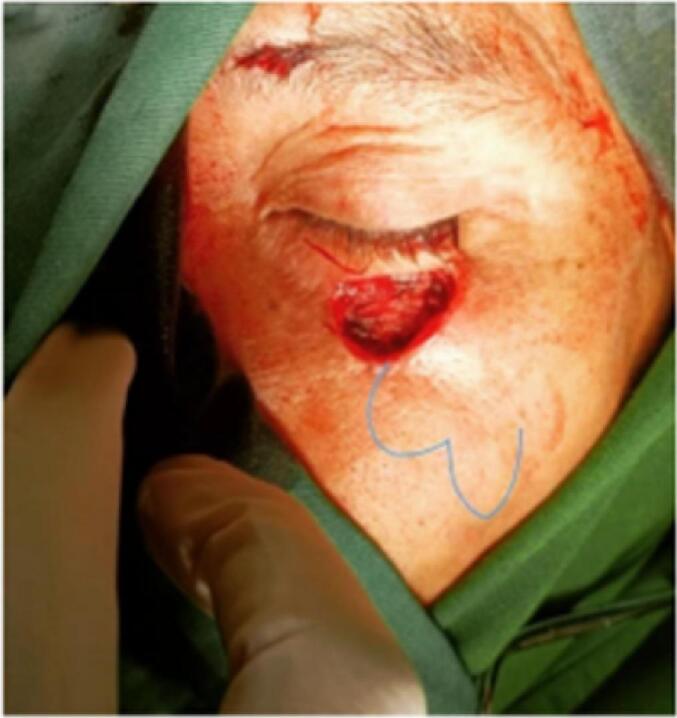
The surgical procedure.

Considering that the pathological mass was oval, the surgical procedure used is the bilobed flap technique with general anesthesia. A center point is selected from the edge of the pathological tissue, and a tangent is drawn towards the edge of the tumor circle. This is intended to prevent the dog's ear from forming on the edge of the tumor. Then the first primary lobe is drawn at a 45-degree angle from the pivot point. The length of the flap is similar to or greater than the tumor (Fig. 2).

Then a second lobe is made at a 90-degree angle from the center point. This second lobe should be longer than the first and narrow at the base so that it is half the diameter of the primary lobe. The secondary lobe is then excised with the ends in a conical shape. This creates a linear scar at the donor site. After excision, the flap is rotated 45°. This is to ensure that the primary lobe covers the defect and the secondary lobe covers the space left. Then the donor site is closed with sutures, and the primary lobe is sutured to the defect. The secondary flap was trimmed and sutured to a left-out flap. Propylene dressings with adhesive tape were then placed for at least one week. The patient has no complaints six months after the surgery.

## 3 Clinical discussion

The main management for ES is surgical resection. Cosmetic reconstruction can be done by tissue expansion and transfer of adjacent tissue, but the prognosis is poor due to local recurrences and malignant transformation [2]. Our patient underwent a bilobed flap technique for surgical resection of the ES. This technique utilizes double transposition flaps. This technique preserves the texture and color of facial skin, minimizing complications in the donor site, provides excellent tissue match, and is a relatively fast procedure due to the single-stage flap thinning performed at the time of insertion [4,5].

In the bilobed flap technique, the double transposition flap in the first lobe fills the primary defect, while the second lobe fills the defect vacated by the first (“secondary defect”). This approach distributes tension over a wider area of cheek tissue but with additional incision length and scarring in a complex curved pattern that makes concealment within the boundaries challenging. The blood supply to the bilobed flap arises from the subdermal plexus of the cheek and differs from the “axial” blood supply which is derived from the vessels entering the flap. Therefore, most of the bilobed flap is raised in the subdermal plane, ensuring that the subdermal vascular plexus remains intact. Venous drainage flows through the subdermal plexus as well, but is sometimes less reliable than arterial flow, making flap congestion theoretically more common. Lymphatic drainage can also be problematic, and the part hanging from the flap will often swell so that its skin surface rises above the surrounding tissue. However, both these phenomenons were not found in this patient. Patients also did not have any complaints, even after six months follow-up period. These findings showed the potential of the bilobed flap technique for eccrine spiradenoma [5,6].

## 4 Conclusion

ES is a benign tumor that resembles many other dermal lesions, requiring proper identification. Considering that its most common predilection is at the facial region, cosmetic aspects must be considered regarding its management. The bilobed flap technique is a surgical management method that showed good potential to treat ES.

## Funding

This study did not receive any funding.

## Ethical approval

No ethical approval has been given, considering that this article is a single case report.

## Consent

Written informed consent was obtained from the patient for publication and any accompanying images. A copy of the written consent is available for review by the Editor-in-Chief of this journal on request.

## CRediT authorship contribution statement

1. Muhammad David Perdana Putra: Concepts and design of study, study investigation and follow up, and manuscript writing.
2. Gumilar Fardhani Ami Putra: Concepts and design of study, study investigation and follow up, and manuscript writing.
3. Widyanti Soewoto: Study investigation and follow up, and manuscript writing.
4. Kristanto Yuli Yarso: Study investigation and follow up, and manuscript writing.
5. Galih Aktama: Study investigation and follow up, and manuscript writing.
6. Shinta Andi Sarasati: Study investigation and follow up, and manuscript writing.

## Declaration of competing interest

There is no conflict of interest.
